# Editorial

**Published:** 1876-11

**Authors:** 


### Editorial.

### THE OHIO STATE DENTAL SOCIETY.

As will be seen on another page, the annual meeting of this body will take place on Wednesday, Dec. 6th, prox. We hope there will be a large gathering of the profession of this state on that occasion. The meetings of this society are always fraught with interest and profit to those who attend; and those who do not attend always lose more than they imagine.

There is always prestige in numbers, and on occasions like this, especially so, if each and every member contributes something of interest. Every one should come to learn, and to teach as well, to some extent at least. Through the influence and operations of such societies as this, is the profession largely indebted for its rapid growth and progress, and any one who enters heartily into the work is a praiseworthy factor therein.

Let every member be present, as there is much work to be done, new modes and processes in practice to be presented and considered. An examination of the new and improved instruments and appliances exhibited at these meetings, is of itself far more than a compensation for the time and money expended in attending. The opportunity to ask questions and receive suggestions upon difficult points of practice, is a privilege that should not be lightly esteemed.

A sense of gratification for what has been accomplished by this, our state dental society, should prompt every member to be present, and the fact that each year, its sphere of usefulness and efficiency for good will be extended and increased, if properly fostered, should be a stimulus to every dentist in the state who has a proper sense and appreciation of our profession's honor and progress. Now let there be such a gathering at this meeting in December as has never been witnessed at any of its annual convocations hitherto. A most cordial invitation is extended to all members of the profession in other states, to be present so far as possible.

Come one and all, and make it a grand time.

---

### MICRO-PHOTOGRAPHS IN HISTOLOGY. NORMAL AND PATHOLOGICAL.

BY CARL SEILER, M. D., IN CONJUNCTION WITH J. GIBBONS HUNT, M. D., AND JOSEPH G. RICHARDSON, M. D.

The object of this publication, as stated in the prospectus, is to replace the microscope, as far as possible, for those physicians (and dentists too) who have neither opportunity nor leisure to make observations with the instrument for themselves; and also to furnish microscopists, for comparison, correct representations of typical specimens in the domain of normal and pathological histology.

The pictures are obtained directly from the microscopic objects by means of photography, and printed from the negative by a reliable mechanical process, and they have the advantage of being faithful copies of the pictures formed by the lens.

It is proposed to give in each monthly issue, pictures of at least one pathological and three normal specimens, to illustrate the difference between healthy and diseased structures.

This is the first time that a systematic publication of micro-photographs has been undertaken, and the work can not fail to be useful, not only to the general practitioner, but also to the scientific world at large.

It is a very valuable work to all who are in any wise interested in the study of the development, growth and structure of animal tissues. It ought to be in the hands of every practitioner and student of dentistry.

The work is published in London by Macmillan & Co., and also by J. N. Coates, Philadelphia.

It is issued monthly.

### EXAMINATIONS.

The annual meeting of the Ohio State Board of Dental Examiners will be held at the American House, in Columbus, on Tuesday, December 5th, at 12 m.

Let all interested take notice and be promptly on hand at that time, as the work of the Board must be completed before the meeting of the State Dental Society on the next day.

### TO MAKE LEACHES BITE.

The "*Doctor*" gives the following from a French medical journal: Place the leaches in a glass half full of cold water, and having carefully cleansed the part with hot water, apply the glass rapidly to the skin. The leaches attach themselves to the diseased part with surprising rapidity.

It seems to the patient that he has received but a single bite.

When all the leaches have bitten, the glass should be raised with caution, so as not to wet the patient needlessly. To do so a sponge or towel should be applied so as to receive the water as it falls out.

If the point to which the leaches are to be applied is very limited, place a sheet of strong paper, having a hole of the size of the part, on the glass.

### OHIO STATE DENTAL SOCIETY.

The Eleventh Annual Meeting of this Society, will be held in the City Council Chamber at Columbus, on Tuesday the fifth day of December, 1876, commencing at ten o'clock a. m., and continuing its session three days.

ORDER OF BUSINESS.

1. Calling of the Roll.
2. Payment of Dues.
3. Reading of the Minutes.
4. Reports of Standing Committees.
5. Reports of Special Committees.

Election of Members at any convenient time during the session, upon report of committee.

Exhibition of Instruments and Appliances, from two to three o'clock p. m., second day.

Election of Officers during the Evening Session of the second day.

SUBJECTS FOR DISCUSSION.

1. Reflex influence from Diseased Teeth.—In what direction and to what extent do they occur?
2. Treatment of the Teeth during gestation and lactation.
3. Anæsthetics.—To what extent is their use justified in the practice of Dentistry?

4. Mechanical Dentistry.—Celluloid, its advantages and disadvantages. Different methods of mounting artificial teeth on metallic bases, and their respective advantages.

5. Dental Education.—Can the dental student best pursue his studies in colleges which are distinctly dental, or in institutions in which both medicine and dentistry are taught?

6. Dental Caries.—General and specific causes and treatment.

7. Do different metals in the mouth cause galvanic action? and, if so—what is the effect upon the teeth and fluids of the mouth?

Col. Blount, the landlord of the American Hotel, offers to entertain members to the Ohio State Dental Society, at two dollars a day, provided the majority of the members stop at the American.

A full attendance is urged upon the members, and every respectable practitioner of dentistry is most cordially invited to be present, and shall be made welcome as a visitor.

F. H. Rehwinkel, J. B. Beauman, and C. R. Butler, Executive Committee.

### STATE BOARD OF DENTAL EXAMINERS.

The State Board of Dental Examiners will hold a meeting at the American Hotel, on Tuesday, the fifth day of December, 1876, at 12 o'clock, m. Candidates for examination will please take notice. J. TAFT, Pres. of Board.

W. P. HORTON, Secretary.

